# Thermal tolerance and climate warming sensitivity in tropical snails

**DOI:** 10.1002/ece3.1785

**Published:** 2015-12-02

**Authors:** David J. Marshall, Enrico L. Rezende, Nursalwa Baharuddin, Francis Choi, Brian Helmuth

**Affiliations:** ^1^ Environmental and Life SciencesFaculty of ScienceUniversiti Brunei DarussalamGadongBE1410Brunei Darussalam; ^2^ Department of Life SciencesUniversity of RoehamptonHolybourne AvenueLondonSW15 4JDUK; ^3^ School of Marine Science and Environmental StudiesUniversiti Malaysia TerengganuTerengganu21030Malaysia; ^4^ Department of Marine and Environmental Sciences and School of Public Policy and Urban AffairsNortheastern UniversityBostonMassachusetts02115

**Keywords:** Climate change, gastropods, global warming, heat coma temperature, mangroves, thermal safety margins, upper lethal temperature

## Abstract

Tropical ectotherms are predicted to be especially vulnerable to climate change because their thermal tolerance limits generally lie close to current maximum air temperatures. This prediction derives primarily from studies on insects and lizards and remains untested for other taxa with contrasting ecologies. We studied the HCT (heat coma temperatures) and ULT (upper lethal temperatures) of 40 species of tropical eulittoral snails (Littorinidae and Neritidae) inhabiting exposed rocky shores and shaded mangrove forests in Oceania, Africa, Asia and North America. We also estimated extremes in animal body temperature at each site using a simple heat budget model and historical (20 years) air temperature and solar radiation data. Phylogenetic analyses suggest that HCT and ULT exhibit limited adaptive variation across habitats (mangroves vs. rocky shores) or geographic locations despite their contrasting thermal regimes. Instead, the elevated heat tolerance of these species (HCT = 44.5 ± 1.8°C and ULT = 52.1 ± 2.2°C) seems to reflect the extreme temperature variability of intertidal systems. Sensitivity to climate warming, which was quantified as the difference between HCT or ULT and maximum body temperature, differed greatly between snails from sunny (rocky shore; Thermal Safety Margin, TSM = −14.8 ± 3.3°C and −6.2 ± 4.4°C for HCT and ULT, respectively) and shaded (mangrove) habitats (TSM = 5.1 ± 3.6°C and 12.5 ± 3.6°C). Negative TSMs in rocky shore animals suggest that mortality is likely ameliorated during extreme climatic events by behavioral thermoregulation. Given the low variability in heat tolerance across species, habitat and geographic location account for most of the variation in TSM and may adequately predict the vulnerability to climate change. These findings caution against generalizations on the impact of global warming across ectothermic taxa and highlight how the consideration of nonmodel animals, ecological transitions, and behavioral responses may alter predictions of studies that ignore these biological details.

## Introduction

Predicting how populations and communities respond to climate change is a foremost concern of global change biologists. Ectothermic animals are considered particularly susceptible to environmental change because their body temperatures and thus physiological performances vary acutely with environmental conditions. While climatic data show that rates of increase in land and sea surface temperatures are generally more pronounced at high latitudes (e.g., Serreze and Barry 2011), there is very high spatial and temporal heterogeneity in the velocity of environmental change (Lima and Wethey 2012). Nevertheless, most comparative studies have suggested that tropical species are at greater risk than temperate (mid‐latitudinal) species, because their upper thermal limits are assumed to lie closer to the maximum environmental temperature (Deutsch et al. 2008; Tewksbury et al. 2008; Huey et al. 2012; Sunday et al. 2012). Even though this prediction is intuitively compelling and has received substantial empirical support, other studies have suggested that consideration of habitat heterogeneity (Bonebrake and Deutsch 2012; Kearney et al. 2012), thermoregulatory behavior (Kearney et al. 2009; Sunday et al. 2014), and temperature variation (Bozinovic et al. 2011; Clusella‐Trullas et al. 2011; Kingsolver et al. 2013; Overgaard et al. 2014; Vasseur et al. 2014) may give rise to remarkably different scenarios. Importantly, most mechanistic work to date has focused on terrestrial insects, lizards, and amphibians (Deutsch et al. 2008; Tewksbury et al. 2008; Dillon et al. 2010; Duarte et al. 2011; Huey et al. 2012), and therefore, observed patterns are limited both from a taxonomic and an ecological perspective.

Here, we study how heat tolerance varies across tropical snails (Class Gastropoda) in intertidal environments, which provide a very different model to investigate the potential impact of ongoing global warming in tropical organisms. Intertidal ecosystems are contrastingly different from most tropical environments because they exhibit enormous thermal variability in time and space, with most of the variation occurring within daily and tidal cycles (Helmuth et al. 2002). Consequently, average temperature estimates poorly encapsulate the habitat thermal heterogeneity and total range of body temperatures that organisms encounter on a regular basis, which can be much higher than the temperature of the surrounding air, even under moderate levels of solar radiation (Helmuth et al. 2002; Marshall et al. 2010, 2013; Gunderson and Leal 2012; Buckley et al. 2013; Logan et al. 2013; Potter et al. 2013; Munoz et al. 2014). In such a spatially and temporally heterogeneous thermal environment, selection associated with temperature extremes is expected to play a major role, and behavioral thermoregulation may constitute a crucial strategy to ameliorating the impact of stressful temperatures (Kearney et al. 2009, 2012; Bonebrake and Deutsch 2012).

Snails are ideal organisms for studying the impacts of climate change for several reasons. They belong to the second most diverse animal phylum (Mollusca) in terms of described species, and although well represented in tropical forests (Schilthuizen and Rutjes 2001; Schilthuizen et al. 2002), their diversity is rapidly declining (Lydeard et al. 2004). From an ecological perspective, snails are outstanding in their ability to colonize highly contrasting habitats. Although predominantly marine, their transition across marine, freshwater, and terrestrial domains is unique among larger metazoans (Dayrat et al. 2011; Webb 2012), and closely related species within lower level lineages (Family) are often found in more than one domain (Frey 2010; Strong et al. 2011; Reid et al. 2012). From a physiological perspective, snails differ fundamentally from ectotherms such as insects, lizards, crustaceans and fishes, because of their limited ability to move in response to changing temperatures (Marshall et al. 2011, 2013). Whereas a close correspondence between thermal tolerance range and the thermal breadth for locomotion is expected in highly active organisms (Angiletta 2009), snails are prone to desiccation during activity and opt instead to withdraw into their shell and remain inactive at high and potentially stressful ambient temperatures. As a result, they can tolerate a significant range of temperatures when inactive (Artacho and Nespolo 2008; Marshall and McQuaid 2011; Marshall et al. 2011). Subsequently, while an ecologically important taxon, more in‐depth comparative studies are necessary to disentangle the role of phylogenetic history and adaptive variation as determinants of heat tolerance (see McMahon 2001) and to assess their resilience to increasing environmental temperatures.

**Figure 1:**
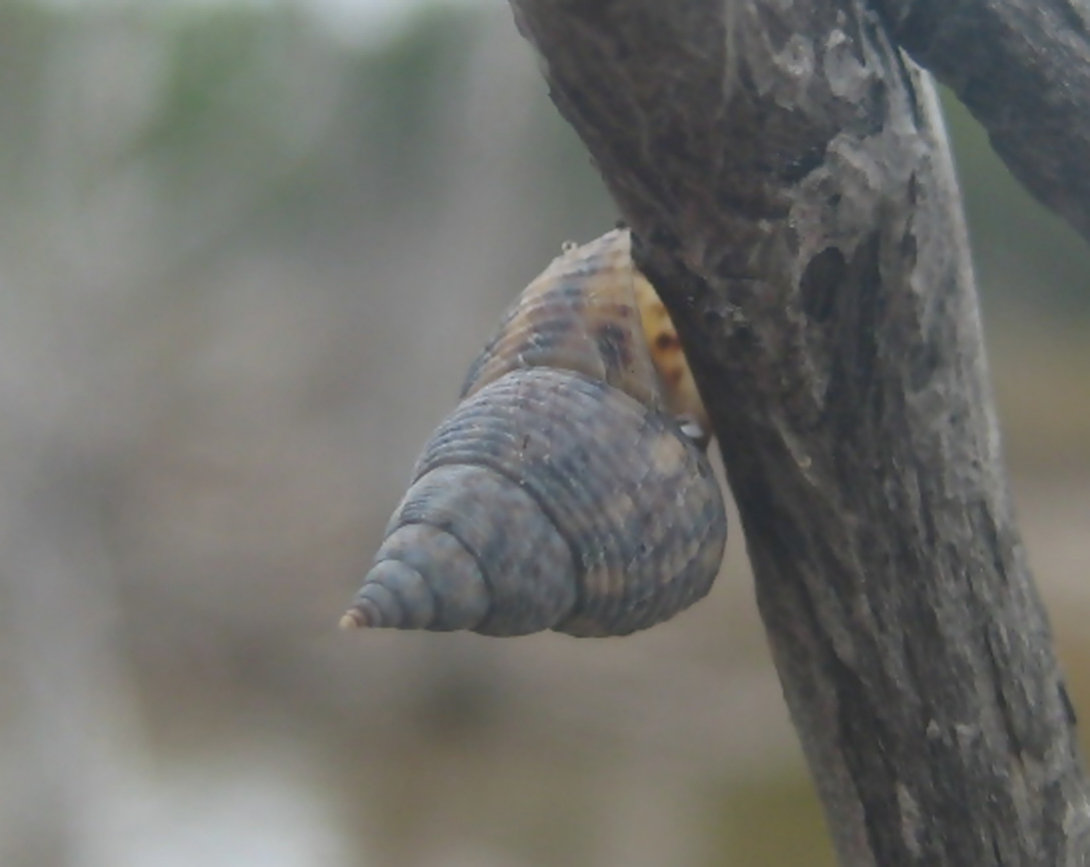
Littorinid snail aestivating on mangrove tree branch.

We focus on two widespread upper‐shore and supratidal gastropod families, Littorinidae and Neritidae (Fig. 1), which prominently display eco‐evolutionary transitions between hot rocky shores and comparatively cool, shaded mangrove forests (Late Cretaceous; Ellison et al. 1999 Frey 2010; Reid et al. 2012). These snails are obligate air‐breathers spending most of their lifetime in air, with the mangrove species encountering similar thermal environments to forest‐dwelling insects, lizards, and amphibians (Frey 2010; Reid et al. 2012). Based on empirical heat tolerance measurements from a global dataset and body temperature predicted with a heat budget model, we specifically address (i) whether there is evidence of adaptation in heat tolerance across geographic locations and/or associated with ecological transitions from rocky shores to mangroves, (ii) the putative role of behavioral thermoregulation to ameliorate the impact of extreme temperatures in these habitats, and (iii) how TSMs (thermal safety margins), and consequently the vulnerability to ongoing climate warming, differ across species, habitats, and localities.

## Materials and Methods

### Thermal tolerance

**Figure 2:**
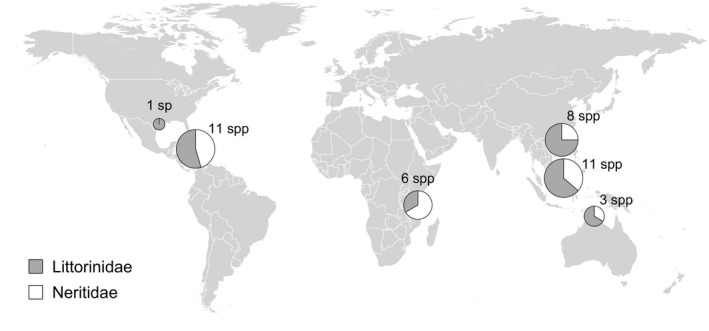
Locations included in this study and their sample size separated by family. From South to North: Australia (12° 30′ S, 130° 36′ E), Tanzania (6° 44′ S, 39°10′ E), Brunei (4° 58′ N, 115° 01′ E), Jamaica (18° 27′ N, 77° 12′ W), Hong Kong (22° 27′ N, 114° 19′ E), and Texas, USA (28° 50′ N, 95° 30′ W). With the exception of Texas, all locations are located within the tropics.

Thermal tolerance of globally distributed tropical snail species of the families Neritidae and Littorinidae (Fig. 2), occupying rocky shore or mangrove habitats, was sourced from the primary literature (Stirling 1982; McMahon 2001; Frey 2010; Reid et al. 2010; Table S1). We also experimentally determined the thermal tolerances of nine species occurring in Brunei Darussalam (4°30′N, 114°40′E, Table S1). These snails mostly inhabit the upper intertidal zones and eulittoral fringes (uninfluenced by regular tidal inundation) and can survive prolonged or near‐continuous aerial exposure, only requiring wetted surfaces for reproduction and/or feeding. They are therefore considered “terrestrial” in terms of their thermal biology and make valid comparators with other terrestrial ectotherms. Maximum body temperatures are typically experienced during daytime resting periods or when aestivating (withdrawn into the shell), when levels of solar radiation and air temperature are at a maximum.

Both HCT (heat coma temperature, the physiological limit to muscle action) and acute ULT (upper lethal temperature) under constant heating were assessed, because snail mortality under field conditions is not determined by a single thermal limit, but instead lies within the range limited by HCT and ULT, varying with exposure period (Denny et al. 2011; Marshall et al. 2011; Woodin et al. 2013; see also Rezende et al. 2014). Locomotion in snails typically stops at temperatures below HCT, allowing a narrow thermal margin in which to adjust shell posture before physiological incapacity for all movement is reached (Marshall et al. 2011; Marshall and Chua 2012). More importantly, HCT marks the induction of a heat shock response, resulting in substantial elevation of resting energetic costs (Marshall et al. 2011; Verberk et al. 2015). This can be potentially lethal during a single prolonged exposure event, but the accumulation of an energy deficit over subsequent days could be also be lethal or have fitness consequences (Marshall et al. 2011; Woodin et al. 2013). Thus, whereas in highly active ectotherms HCT has been interpreted as the point of “ecological death” (e.g., Cowles and Bogert 1944; Mora and Opsina 2002), locomotor performance in snails is often impaired at temperatures substantially lower than ULT to prevent water loss through evaporation. Where thermal tolerance measurements differed among sources for the same species due to seasonal or locality differences, we used the lowest (i.e., most conservative) value. Data for all of the Brunei species were included in the analyses regardless of whether other records for the same species already existed. A total of 40 species/localities were used in the statistical and phylogenetic analyses (Fig. 2; Table S1).

Upper lethal temperatures were assessed from the flatline (or endpoint) temperature for cardiac function (Marshall and McQuaid 2011; Marshall et al. 2011). Following collection from nearby rocky shores and mangroves in Brunei, snails (*Nerita balteata, Nerita planospira, Echinolittorina malaccana, Echinolittorina vidua, Littoraria carinifera, Littoraria lutea, Littoraria pallescens, Littoraria strigata/articulata,* and *Littoraria undulata*) were kept in laboratory aquaria for 2 days in high humidity air at 26 ± 1°C (temperature near the daily minimum). Prior to experiments, they were rinsed and completely rehydrated in seawater collected from near their habitat. Shells were towel dried and fitted with infrared optoelectronic sensors (CNY70; Vishay Semiconductors Shelton, CT, USA) near the mantle cavity using BluTac (Bostick Australia). Experiments were performed in chambers of high humidity (>80% R.H.; 250 or 500 mL beakers) held inside a Peltier‐cooled incubator (Schwabach Memmert, IPP400, Germany). Individual snails were heated at an ecologically relevant rate (0.25°C/min) between 25 and 60°C, and heart rate was recorded every 1 min. Heart beat signals from the sensors were filtered, amplified, and digitally logged (Castle Hill custom built preamp; PowerLab/4SP, Chart v5, ADInstruments, Australia). Operative snail temperature was monitored simultaneously with heart rate using fine K‐type thermocouples (Omega, Stamford, CT, USA) (inside a mimic shell) and Fluke 54 Series II digital thermometers (Fluke, Washington).

The temperature at which physical detachment occurred in heated seawater‐filled tubes was defined as HCT (McMahon 2001). Submerged snails (17–30 per species as above) that had adhered to the sides of 50‐mL or 250‐mL glass tubes were exposed in a water bath (Grant W28, UK) to 5°C increments every 20 min between 30 and 40°C, then to 1°C increments every 10 min above 40°C. The number of tubes with detached snails was recorded with each temperature change. Water temperature was continuously monitored using K‐type thermocouple sensors connected to Fluke thermometers.

### Environmental temperatures

Environmental temperatures in Brunei Darussalam were recorded using dataloggers (I‐button, Thermochron, DS1921G Maximum Integrated, San Jose, CA) contained within white plastic capsules, attached to the substratum. Temperatures were simultaneously recorded in mangrove and rocky shore habitats every 10 min for 30 days during the hottest time of the year (22 April–24 May 2013). Unlike rocky shore snails, which rest in both sunned or shaded habitats (Marshall et al. 2010, 2013; Marshall and Chua 2012), mangrove forest snails behaviorally select relatively cool, shaded resting sites (typically below 35°C, Chapperon and Seuront 2011). Consequently, dataloggers were deployed on the sun‐exposed eulittoral fringe zone of a rocky shore (Jerudong Park Medical Centre, N 04°57′02.7″, E 114°49′44.6″, *N* = 3) and shaded undersurfaces of mangrove leaves (Pulau Bedukang, N 04°58′40.3″, E 115°03′49.3″, *N* = 3 replicates), which correspond to the hottest habitat in each environment. To estimate thermal heterogeneity in body temperatures associated with these environments (Helmuth and Hofmann 2001; Helmuth et al. 2006; Mislan et al. 2009; Marshall et al. 2013), additional recordings were taken for briefer periods on shaded rock surfaces (*N* = 3, 12 day, 20 July–01 August 2012, Marshall et al. 2013), on the mud surface under mangrove trees, and at the base of a mangrove tree (these records represent the combined effects of aerial conditions, solar radiation, and tidal inundation; *N* = 1 each, 42 days, 24 April–04 June 2012). Although the recordings based on the most extreme rocky shore habitat occupied by *E. malaccana* might not be entirely representative of the thermal regimes of other species lower on the shore, the maximum temperatures experienced should be similar among all species of similar size and roughly comparable morphology, assuming mid‐day solar exposure (Helmuth et al. 2002).

Long‐term (20 year) estimates of body temperature in shaded and sun‐exposed habitats across geographic localities were calculated using a simple heat budget model modified from one developed to calculate temperatures of mussels (Helmuth 1998; Helmuth et al. 2011). The fundamental is based on a steady state model of heat flux to estimate body temperature presented first in Helmuth (1998) and adjusted in Kearney et al. (2010) and Helmuth et al. (2011). The model was modified by changing the size of the animal and amount of contact with the substratum to represent a generic snail. Body temperatures were calculated using environmental data extracted from the National Centers for Environmental Prediction Climate Forecast System Reanalysis (CFSR; Saha et al., 2010). CFSR provides hourly data at a global resolution of 38 km over the period of 1979–2009. Hourly air temperature, wind speed, and solar radiation data from 1989 to 2009 were downloaded for the pixel closest to each study site from which we obtained physiological tolerance data from the literature. A generic body size of 1 cm was used for all species, and no attempt was made to account for small variations in morphology (which were expected to have a minimal impact on body temperature). A fixed cloud cover of 60% was used to account for downwelling infrared radiation, and wind speed was set with a basement of 10 cm/sec to account for free convection (Helmuth 1999). Results were additionally compared against model outputs from a modified land surface model (Mislan and Wethey 2015) and body temperatures calculated from datalogger recordings in Brunei Darussalam, employing a linear regression model (*T*_e_ = 3.62 + 0.87 iButton temperature; *R* = 0.94; *P* < 0.001, *n* = 320) based on simultaneous 1 min measurements from an iButton and an adjacent mimetic *E. malaccana* exposed to direct sunlight for 10 h (K‐type thermocouples, Fluke 54 Series II digital thermometers). Estimates were found to be similar, with mean daily maxima differing by little more than 1°C between our model and values based on datalogger recordings. To study the potential impact of extreme climatic events on tolerance limits, we employed the maximum body temperature estimated across the 20‐year period in subsequent analyses, which typically occurred during maximum air temperatures coupled with maximum solar radiation.

### Thermal safety margins

In previous studies, several temperature indexes, including mean annual temperature, mean daily maximum temperature during the warmest 3 months of the year, and the mean temperature during the three warmest months of the year, have been compared against physiological limits to estimate resilience to climate change (Deutsch et al. 2008; Huey et al. 2009; Diamond et al. 2012; Kellermann et al. 2012; Logan et al. 2013). Similarly, calculations of TSMs often involve the difference between optimal performance temperatures and mean environmental temperatures (Deutsch et al. 2008; Huey et al. 2009). However, in the case of snails, the limit to locomotor performance (HCT) and the thermal tolerance limits (ULT) are often very different. Consequently, TSMs were calculated as the difference between tolerance limits (HCT or ULT) and maximum body temperatures estimated at each site for completely shaded and exposed habitats, which encompass the total range of variation resulting from different levels of solar exposure (e.g., differences in slope, orientation or shade cover across microhabitats). Solar radiation is expected to have a major impact on body temperature in species or populations inhabiting rocky shores, but not mangroves, and therefore, we assumed that the effects of solar exposure were negligible in the latter. Values of TSM approaching zero indicate a high vulnerability to increased temperatures, and, from these analyses, we were able to estimate which species have faced thermally restrictive conditions during the 20‐year period for which environmental records are available and how thermal selection varies across lineages, habitats (i.e., mangrove vs. rocky shores), and geographic locations.

### Phylogenetic analyses

To compare thermal limits and safety margins among the families and habitats, we built a dated phylogeny for the snail species (*N* = 34). Phylogenetic relations and divergence times for Neritidae and Littorinidae were obtained from Frey and Vermeij (2008) and Reid et al. (2012), respectively. The divergence time between these families was set to 470 Mya according to the stratigraphic ranges of main gastropod groups in the fossil record (fig. 10.11 in Fryda et al. 2008). Divergence times between populations of *Nerita albicilla* from Tanzania and Hong Kong and *Nerita chamaeleon* from Brunei and Hong Kong corresponded to 5.0 and 8.0 Mya, respectively, based on estimates provided by Frey and Vermeij (2008). In the absence of similar information for *N. balteata*, divergence times between populations collected at Brunei and Darwin, Australia, were assumed to be 5.0 Mya.

We assessed whether habitat exhibits phylogenetic conservatism by comparing the minimum number of transitions necessary to reconstruct the observed dataset against a null distribution, obtained after randomizing habitat character states 999 times across the tips of the phylogeny (the *Fixed Tree, Character Randomly Reshuffled* model in Madisson and Slatkin 1991; see also Rezende and Diniz‐Filho 2012). The number of transitions in each replicate was calculated with parsimony employing an ad hoc code in R (code available from E.L. Rezende upon request). Subsequently, we employed a phylogenetic multiple regression and a model comparison approach to determine whether HCT and ULT exhibited phylogenetic signal and differed across habitats and families. These analyses were mathematically equivalent to an ANOVA comparing averages between families (Neritidae vs. Littorinidae) and/or habitats (rocky shores vs. mangroves), accounting for phylogenetic effects including a matrix of variance–covariance in the residual error structure of the generalized‐least squares regression (Grafen 1989; Garland and Ives 2000; Rohlf 2001; Revell 2010; Rezende and Diniz‐Filho 2012). We considered three different error structures, assuming a Brownian model of evolution (in which the expected phenotypic divergence is proportional to divergence time), a model in which branch lengths are optimized as a function of parameter *λ* to maximize the match between phenotypic divergence and divergence times (Pagel 1999) and a star phylogeny in which phylogenetic signal is absent. Because *λ *= 1 corresponds Brownian motion, and *λ *= 0 corresponds to a star phylogeny, these three error structures ultimately assess whether the phenotypic data exhibit a hierarchical structure corresponding to species' phylogenetic history.

Models were compared employing Akaike's information criterion (AIC), a model selection approach formally linked to maximum likelihood theory in which, the lower the AIC estimate, the higher the fitness of the model (Burnham and Anderson 2002; Turkheimer et al. 2003). Given the small sample size, we employed the small sample formulation AIC_c_ to quantify the fitness of each model and Akaike weights (*w*_*i*_) to estimate the weight of evidence in favor of each model within the set. Succinctly, *w*_*i*_ provides the probability of model *i* being the correct one given all the models tested.

## Results

### Thermal tolerance

**Figure 3:**
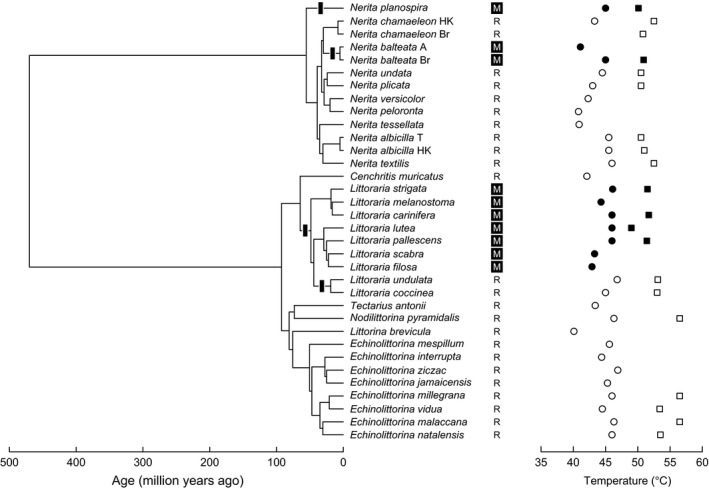
Phylogeny for Neritidae and Littorinidae employed in analyses, with the transitions between rocky shores (R) and mangroves (M) highlighted by the black rectangles. Habitat condition, heat coma temperature (circles), and upper lethal temperatures (squares) are superimposed on the phylogeny.

Parsimony analyses show that only 4 habitat transitions are necessary to reconstruct the empirical dataset (Fig. 3), whereas the minimum number of transitions obtained in the 999 randomized replicates was 9. Habitat is therefore highly conserved in the phylogeny (*P *<* *0.001), which poses an important statistical challenge from a comparative perspective because of the inherently low statistical power to detect habitat effects. For instance, a sign test designed to test whether species inhabiting mangroves have a lower thermal tolerance than their counterparts from rocky shores can, with only 4 transitions, support this hypothesis in the best‐case scenario with a *P *= 0.5^4^ or 0.0625. Thus, even if lowered thermal tolerance was observed in all ecological transitions to mangroves, it is impossible to reject the null hypothesis with a *P *≤* *0.05. Because habitat and family are highly confounded with the error structure included in the regression models, these models are not mutually exclusive and results must be considered as a whole.

**Figure 4:**
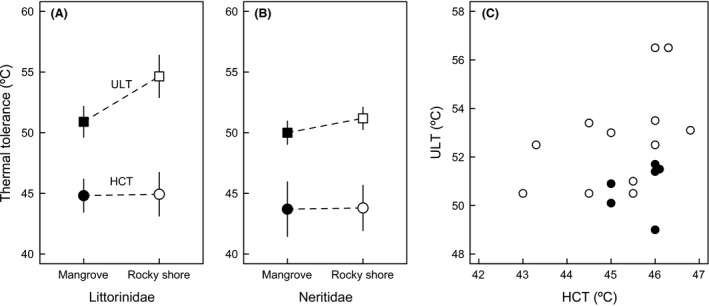
Thermal tolerance across families and habitats. HCT (heat coma temperature) and ULT (upper lethal temperatures) (least square means ± SE) are shown for (A) littorinid and (B) neritid snails in rocky shore and mangrove habitats. (C) Relationship between ULT and HCT for those species in which both estimates are available (open symbols = rocky shore, closed symbols = mangroves).

Estimates of HCT ranged between 40.1 and 46.9°C, and ULTs between 49.0 and 56.5°C (Fig. 3; Table S1). Analyses of the ecological and phylogenetic correlates for HCT and ULT provided contrasting results (Table 1). For HCT, AIC_c_ and *w*_*i*_ results provide little support for phylogenetic signal (the cumulative *w*_*i*_ for models with *λ *= 0 corresponds to 0.66) and suggest that this trait is higher in Littorinidae than in Neritidae (Table 1; Fig. 4). For instance, models including family have a cumulative *w*_*i*_ of 0.85 and this factor is statistically significant in models assuming *λ *= 0. Conversely, this factor is not significant in analyses with a phylogenetic structure, suggesting that the large divergence time between these families and a random model of phenotypic evolution can explain observed differences. The model with the best fit (AIC_c_ = 137.05, *w*_*i*_ = 0.32) supports a low phylogenetic signal, a 1.34 ± 0.65°C (±SE) difference in HCT between families (adjusted estimates of 44.9 and 46.3°C for Neritidae and Littorinidae, respectively) and no habitat effects.

**Table 1:** Comparison of regression models testing family and habitat effects on HCT and LT under different evolutionary scenarios (all models include an intercept, not shown for simplicity). *λ *= phylogenetic correlation ranging between 0 (star phylogeny) and 1 (original phylogeny assuming a Brownian mode of evolution), AIC_c_ = Akaike's information criterion for small samples, *w*_*i*_ = Akaike's weight. *P*‐values correspond to a 2‐tailed test

Model	Family	Habitat	*λ*	AIC_c_	*w*_*i*_ a
HCT			0	138.68	0.14
HCT ~ Family	*t*_31_ = −2.038, *P* = 0.048		0	137.05	0.32
HCT ~ Habitat		*t*_31_ = −0.275, *P* = 0.785	0	140.84	0.05
HCT ~ Family + Habitat	*t*_30_ = −2.00, *P* = 0.054	*t*_30_ = −0.11, *P* = 0.91	0	138.54	0.15
HCT			0.23	140.34	0.06
HCT ~ Family	*t*_31_ = −0.15, *P* = 0.88		0.88	138.67	0.14
HCT ~ Habitat		*t*_31_ = −0.193, *P* = 0.84	0.26	141.71	0.03
HCT ~ Family + Habitat	*t*_30_ = −0.14, *P* = 0.89	*t*_30_ = −0.28, *P* = 0.78	0.90	139.76	0.08
HCT			1	149.81	0.00
HCT ~ Family	*t*_31_ = −0.038, *P* = 0.97		1	145.89	0.00
HCT ~ Habitat		*t*_31_ = −0.237, *P* = 0.81	1	149.85	0.00
HCT ~ Family + Habitat	*t*_30_ = −0.04, *P* = 0.97	*t*_30_ = −0.23, *P* = 0.82	1	146.08	0.00
ULT			0	91.16	0.00
ULT ~ Family	*t*_18_ = −2.63, *P* = 0.017		0	86.26	0.00
ULT ~ Habitat		*t*_18_ = 2.22, *P* = 0.039	0	87.60	0.00
ULT ~ Family + Habitat	*t*_17_ = −3.87, *P* = 0.0012	*t*_17_ = 3.52, *P* = 0.0026	0	78.38	0.13
ULT			0.98	83.06	0.01
ULT ~ Family	*t*_18_ = −0.46, *P* = 0.65		0.99	80.52	0.05
ULT ~ Habitat		*t*_18_ = 2.35, *P* = 0.030	0.89	80.65	0.04
ULT ~ Family + Habitat	*t*_17_ = −0.59, *P* = 0.56	*t*_17_ = 2.11, *P* = 0.049	0.94	79.12	0.09
ULT			1	80.71	0.04
ULT ~ Family	*t*_18_ = −0.42, *P* = 0.68		1	77.58	0.20
ULT ~ Habitat		*t*_18_ = 1.63, *P* = 0.12	1	79.16	0.09
ULT ~ Family + Habitat	*t*_17_ = −0.41, *P* = 0.69	*t*_17_ = 1.58, *P* = 0.13	1	76.50	0.34

HCT, heat coma temperature; ULT, upper lethal temperatures; LT, lethal temperature.

a *w*_*i*_ values must add up to 1.0, and only add up to 0.97 and 0.99 here due to rounding issues.

Conversely, regression models for ULT support a strong phylogenetic signal for this trait, with the cumulative *w*_*i*_ for models assuming a phylogenetic structure corresponding to 0.87 (with the remaining *w*_*i*_ = 0.13 supporting a model that includes family as a factor, which inherently involves a phylogenetic component in spite of the assumption of *λ *= 0). For ULT, estimates of AIC_c_ and *w*_*i*_ results support the inclusion of family (cumulative *w*_*i*_ = 0.81) and habitat (cumulative *w*_*i*_ = 0.70) in the model. Importantly, habitat was statistically significant or bordered significance in all models (1‐tailed *P *<* *0.065; note that we predicted that heat tolerance is rocky shores is higher than in mangroves, and therefore, a 1‐tailed test is adequate in this case), in spite of the low statistical power due to the low number of habitat transitions when phylogenetic structure is taken into account. The model with the best fit (AIC_c_ = 76.50, *w*_*i*_ = 0.34) is in accordance with these general conclusions. Taken together, results provide circumstantial evidence that ULT shows limited evolutionary response to ecological transitions between thermally extreme tropical rocky shores and thermally benign mangrove trees, and this response seems to be more pronounced in Littorinidae (Fig. 4). Accordingly, inclusion of the family by habitat interaction improves the model fit (AIC_c_ = 75.40), and in this model, rocky shore species exhibit ULT on average 3.0 ± 1.5°C (±SE) higher than mangrove species (adjusted means of 54.1 and 51.1°C, respectively; *P*hab = 0.065 and *P*interaction = 0.23). Assuming that the observed tolerance in rocky shore species corresponds to the ancestral condition, it seems that lineages that have invaded mangroves exhibit somewhat lower ULT, although it is unclear whether this result reflects evolutionary differences or inducible changes. Importantly, some rocky eulittoral fringe species conserve extraordinary levels of heat tolerance (Marshall et al. 2011). Taken together, these results suggest that both the ecology and thermal physiology of these snails are highly conserved in the phylogeny, with major differences being observed across the two families studied here.

Additionally, analyses indicate that HCT and ULT can evolve to some extent independently from one another, as lower ULTs seem to have evolved in mangrove species without a concomitant decrease in HCT (Fig. 4). Whereas a regular regression between HCT and ULT suggests that these variables are marginally correlated (*t*_19_ = 1.52, 1‐tailed *P *=* *0.073), phylogenetic analyses were never statistically significant (1‐tailed *P *> 0.17 for models assuming Brownian motion or optimizing *λ*). Consequently, even though species with higher HCT tended to exhibit higher ULT in the subset of snails in which both variables were measured, this correlation is explained to some extent by common ancestry. Even though correlated evolution finds limited support in this reduced dataset, it cannot be entirely dismissed because of the inherently low statistical power due to a limited sample size and measurement error.

### Environmental temperatures and TSM

**Figure 5:**
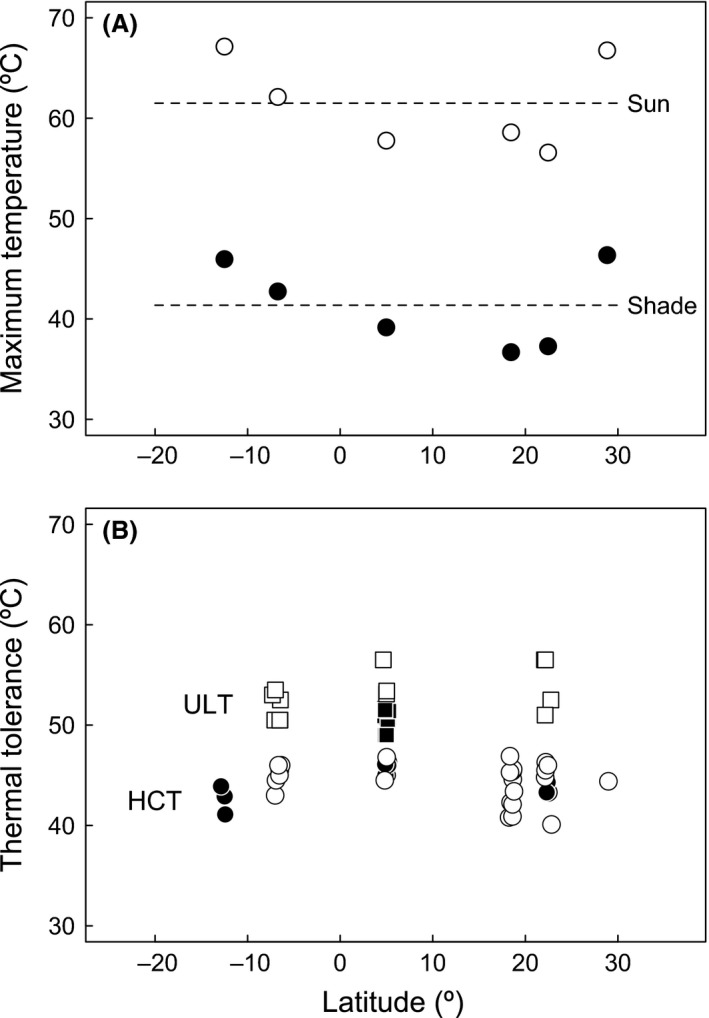
Body temperature and thermal tolerance across locations. (A) Maximum estimated body temperatures for exposed and sheltered habitats and (B) heat tolerance measurements heat coma temperature (circles) and upper lethal temperatures (squares) plotted against latitude, with species inhabiting mangrove and rocky shores shown in black and white, respectively. Geographic locations correspond, from South to North, to Australia, Tanzania, Brunei, Jamaica, Hong Kong, and Texas (see Fig. 1).

Estimated maximum body temperatures within shaded or exposed habitats varied up to 10°C across the six geographic locations studied here, with locations at the extremes of the latitudinal range exhibiting higher temperatures (Fig. 5; Brunei habitat temperatures are given in Fig. S1). Within each location, thermal regimes were strikingly different between shaded and exposed habitats, with solar exposure resulting in an average increase in body temperature estimates of 20.1 ± 1.2°C (±SD). Therefore, temperature extremes across rocky shores versus mangroves can vary substantially more than values observed in similar habitats across widely dispersed geographic locations (Fig. 5). Because temperature estimates are highly asymmetric across hemispheres, and locations are found in very disparate regions, we include geographic location as an independent factor in subsequent analyses and not absolute latitude.

**Figure 6:**
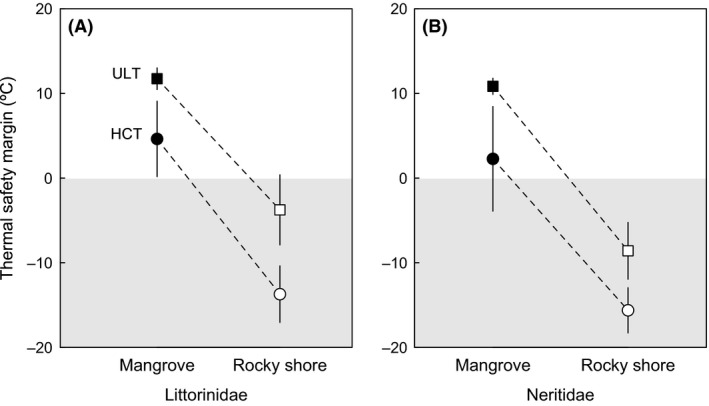
Thermal safety margins (mean ± 95% CI) of littorinid (A) and neritid snails (B) calculated as the difference between HCT (heat coma temperature) and ULT (upper lethal temperatures) and maximum body temperatures expected in mangroves and rocky shores. The gray area indicates the region in which body temperatures surpass heat tolerance estimates, which should result in immobility (HCT) and/or death (ULT).

Thermal safety margins were calculated employing HCT and ULT and maximum body temperatures expected for each species in its habitat. Maximum estimated body temperature in species inhabiting mangroves generally fell below HCT and ULT, and average values of TSMs were 5.1 ± 3.6°C and 12.5 ± 3.6°C (±SD), respectively. In contrast, body temperatures in rocky shore species estimated using the heat budget model consistently surpassed both indexes of heat tolerance, giving rise to negative TSM of −14.7 ± 4.0°C for HCT and −6.4 ± 3.7°C for ULT (Fig. 6). While the maximum temperatures estimated for sunny environments were extreme (~60°C) notably, they were not dissimilar from rock surface temperatures reported in other studies (e.g., Huey et al. 1989; Williams and Morritt 1995). These analyses also suggest that species within Neritidae are more vulnerable to extreme climatic events, not only due to their lower HCT and ULT (Table 1) but also because 5 of 12 species inhabiting rocky shores (~42%) are found in geographic regions with very high temperatures (Australia, Tanzania, or Texas). Conversely, only 3 of 16 species of Littorinidae inhabit rocky shores in these regions (~19%). Accordingly, comparison of AIC_c_ values obtained for different regression models supports variation in TSM emerging from a complex interaction between habitat, geographic location, family affiliation, and phylogenetic history (Table 2).

**Table 2:** Comparison of regression models testing phylogenetic and geographic effects on TSMs. TSM were calculated as maximum body temperature – HCT, where body temperature for species in mangroves and rocky shores correspond to estimates in the shade and exposed to the sun, respectively. All models include an intercept, not shown for simplicity. *λ *= phylogenetic correlation set to 0 (star phylogeny) or 1 (original phylogeny) because regression models adjusting for *λ* did not always converge. AIC_c_ = Akaike's information criterion for small samples, *w*_*i*_ = Akaike's weight. Similar analyses with ULT were not performed because of the small sample size and only three of the six geographic locations being represented

Model	*λ*	AIC_c_	*w*_*i*_
TSM ~ Habitat	0	181.81	0.00
TSM ~ Geography	0	207.77	0.00
TSM ~ Family	0	241.26	0.00
TSM ~ Habitat + Geography	0	134.70	0.01
TSM ~ Habitat + Family	0	178.62	0.00
TSM ~ Geography + Family	0	207.27	0.00
TSM ~ Habitat + Geography + Family	0	132.17	0.03
TSM ~ Habitat	1	211.18	0.00
TSM ~ Geography	1	200.15	0.00
TSM ~ Family	1	228.18	0.00
TSM ~ Habitat + Geography	1	127.77	0.29
TSM ~ Habitat + Family	1	205.42	0.00
TSM ~ Geography + Family	1	195.44	0.00
TSM ~ Habitat + Geography + Family	1	126.05	0.67

HCT, heat coma temperature; TSM, thermal safety margins; ULT, upper lethal temperatures.

## Discussion

**Figure 7:**
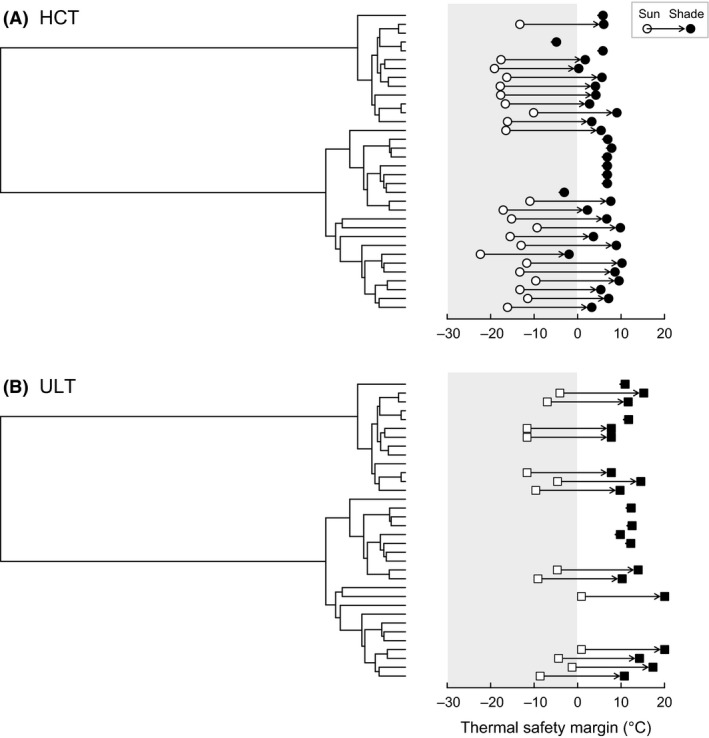
Thermal safety margins (TSM) superimposed on the species phylogeny. The impact of solar radiation was assumed to be negligible for species inhabiting mangroves. The arrows describe the predicted impact of behavioral thermoregulation (i.e., sun avoidance) on TSMs, and the gray area indicates the region in which body temperatures surpass estimates of heat tolerance. During sun exposure, TSM = −14.8 ± 3.3°C and −6.2 ± 4.4°C for heat coma temperature and upper lethal temperatures, respectively, and in the shade TSM = 5.1 ± 3.6°C and 12.5 ± 3.6°C (±SD).

The hypothesis that tropical ectotherms are physiologically vulnerable to future environmental warming because the current temperatures they encounter are already close to their thermal limits has received much empirical support (Deutsch et al. 2008; Tewksbury et al. 2008; Huey et al. 2009, 2012). Additionally, extinction risk predicted by paleontologically calibrated models suggest that coastal regions in the tropics are particularly vulnerable to human activity and climate change (Finnegan et al. 2015). However, mechanistic studies assessing how global warming might impact coastal organisms are currently scarce (but see Kearney et al. 2010; Wethey et al. 2011; Wernberg et al. 2012; Bernhardt and Leslie 2013; and references therein). For snails from intertidal environments, however, our results suggest that the hypothesis that temperatures in the tropics may be restrictive is only partially supported. On the one hand, they indicate that, in spite of an elevated thermal tolerance, virtually all species may be subject to strong thermal selection when exposed to the sun during climatic extremes (Fig. 7). On the other hand, the existence of sheltered microhabitats (crevices and cracks) that facilitate behavioral thermoregulation and varying thermal regimes with tidal inundation may alleviate midday solar exposure (Helmuth and Hofmann 2001; Helmuth et al. 2006; Mislan et al. 2009; Marshall et al. 2010, 2013) and result in reduced mortality even in the extreme circumstances envisioned in our analyses. The risk of mortality therefore is not a simple matter of “habitat temperature” (and especially not just air temperature), but rather a combination of microclimatic conditions coupled with an organism's ability to access them (Kearney 2006). Thus, the evolution of very high thermal tolerances in these intertidal snails, which exhibit ULTs that are generally higher than those of tropical insects or lizards (see Araújo et al. 2013; Hoffmann et al. 2013), seems to emerge from the interplay between two factors: very strong thermal selection during sun exposure combined with the enormous heterogeneity in available microclimates that prevents major population crashes during extreme climatic events. Notably, similar arguments can be made not just for snails but for any terrestrial organism living in at least partly sun‐exposed environments (Kearney et al. 2012). Accordingly, snails of the families studied here, which occupy the high shore zone, are generally capable of resisting climatic abnormalities, evidenced by their resilience to recent extreme natural heat wave events (Williams and Morritt 1995; Harley 2008; Garrabou et al. 2009). Below we discuss how our results contribute to our understanding of how these intertidal gastropods might respond to ongoing global warming.

Analyses provide only circumstantial evidence of physiological adaptation to local thermal conditions (but see McMahon 2001). For instance, differences in heat tolerance observed across geographic locations were not related to extreme thermal conditions, and species with the highest HCT are surprisingly found in locations in which body temperature was less extreme (Fig. 5). Although both HCT and ULT differed significantly between locations in phylogenetic models including only this factor (*P* = 0.042 and *P* = 0.039), maximum body temperature estimated in sheltered or exposed areas was not correlated with HCT or ULT in any of the models tested (*P* > 0.15 in all cases). Similarly, the colonization of mangrove habitats did not have a major influence on heat tolerance in spite of the prominent differences in thermal environments observed across mangrove forests and rocky shores. The lower ULT in Littorinidae inhabiting mangroves provides some support to physiological adaptation following an ecological transition or might, alternatively, reflect the capacity of *Echinolittorina* snails to withstand the extreme temperatures of rocky eulittoral fringe habitats (Fig. 3; see also Marshall et al. 2010; Marshall and McQuaid 2011; Marshall et al. 2011). However, the average difference of ~4.0°C in ULT of species from different habitats, which could well reflect inducible changes rather than evolutionary differences with a genetic basis, remains relatively small when compared against the 20°C difference in thermal environment between rocky shores and mangroves (Fig. 5). Notably, no differences in ULT were detected across species inhabiting rocky shores and mangroves in Neritidae (Fig. 4).

Results also highlight that heat tolerance is highly conserved in the phylogeny, in agreement with studies on HCT in other gastropods (McMahon 2001) and ULT of terrestrial ectotherms (Clusella‐Trullas et al. 2011; Kellerman et al. 2012; Grigg and Buckely 2013). Different factors may account for the limited physiological responses to contrasting thermal conditions, including a low adaptive potential in heat tolerance (Araújo et al. 2013; Hoffmann et al. 2013), relaxed thermal selection following the colonization of mangroves, and spatial heterogeneity in available microclimates (Wethey et al. 2011). Although our comparative analysis cannot discriminate between these alternatives, estimates of TSM strongly suggest that behavioral thermoregulation and microhabitat use can buffer the impact of solar radiation and thermal selection in most instances (Fig. 7). Importantly, these results are conservative because we have employed the most extreme body temperatures estimated in a 20‐year period, and hence, averaging effects are not an issue and generally populations within this timeframe will not be subjected to the thermal regimes simulated here. Based on HCT and ULT measurements, most species under shelter would cope with an increase in air temperature of 2.5°C (Fig. 7), which would likely lead to an increase in body temperature of a similar magnitude, all other factors remaining constant (Gilman et al. 2006). However, with this type of data, we cannot ascertain how prolonged exposure to sublethal temperatures might impact energy and water balance (see Cook 2001; Dillon et al. 2010; Schneider et al. 2010; Marshall et al. 2011; Rezende et al. 2011). Multiple exposures to sublethal temperatures may have detrimental effects on thermal tolerance and, consequently, on survival in the long term, and this possibility remains to be tested in future studies.

Model comparison indicates that habitat use and geographic distribution are better predictors of vulnerability to climate change in the species studied here than their thermal tolerance (Table 2). Although the model with best fit includes family affiliation, suggesting that species‐specific physiological attributes might be relevant for predictive purposes, regression coefficients show that this effect is relatively small: TSM estimates differ 18.9 ± 1.0°C between habitats, between 2.0 ± 1.6°C and 12.4 ± 0.6°C across locations, and only 1.0 ± 6.3°C between families (±SE). Accordingly, when analyses are repeated removing the effects of solar exposure between habitats – that is, with temperature estimates in the shade for the entire dataset – geographic location still accounts for 81.4% of the variation in TSM (*r* ^2^ obtained from a conventional regression) in spite of the relatively constrained range of latitudinal variation, with all but one species falling within the tropics (Fig. 2). This is not surprising given the low variation in HCT and ULT across species (6.8 and 7.5°C, respectively; Fig. 3) when contrasted against the thermal variation observed within and across geographic locations encompassing 30.7°C (Fig 5A). Consequently, comparisons between the variability in thermal tolerance against the overall variation in environmental conditions can provide a relatively simple diagnostic of how vulnerable species might be distributed across habitats and/or localities. For example, TSM values were consistently lower in Australia, Tanzania, and Texas irrespective of species composition (Fig. 5), suggesting that these communities might be more vulnerable to increasing temperatures. A similar scenario is expected for other intertidal organisms if their heat tolerance, either HCT or ULT, happens to fall within a narrow window as reported here for Littorinidae and Neritidae (Fig. 3).

To summarize, we studied which factors explain the observed variation in heat tolerance across tropical snails inhabiting intertidal environments and found little support for thermal adaptation across habitats and geographic locations. We also assessed whether increasing temperatures predicted by climatic models might threaten these organisms and suggest that, contrary to what is predicted for other tropical organisms (Deutsch et al. 2008; Tewksbury et al. 2008; Huey et al. 2012; Sunday et al. 2012), the direct impact of warming temperatures should be ameliorated by behavioral thermoregulation if suitable shaded microhabitats are available (see Kearney et al. 2009; Marshall et al. 2013; Sunday et al. 2014). This is because in intertidal environments, which exhibit extreme levels of temperature variation, the general expectation that tropical organisms exhibit a narrow window of temperature tolerance may not hold (see Ghalambor et al. 2006). Consequently, our findings offer a caution to the general assumption of climate vulnerability of tropical ectotherms (see Huey et al. 2012) and advocate greater consideration of the diversity of taxonomic and ecological systems in future assessments of climatic sensitivity of tropical communities.

## Data Accessibility

All data used in this manuscript are present in the manuscript and its supporting information.

## Conflict of Interest

None declared.

## Supporting information


**Figure S1.** Operative temperature data for sun‐exposed rocky shore (A, C, E, G) and shaded mangrove habitats (B, D, F, H). Mean temperatures for three habitats recorded every 10 min for 30 days (A, B), and frequency distributions of these means (E, F). (C, D) Average temperature for a 24 h period (0 and 144 = 06h00; 30 day) based on recordings in (A, B) are shown as thin solid lines, and for the hottest temperature in each habitat as thick solid lines. Dashed lines in the rocky shore panel indicate shaded (coolest) habitat, while dashed and dotted lines in the mangrove panel indicate temperatures on the mud surface (under tidal influence) or at the base of a tree, respectively. (G, H) Frequency distributions for daily maximum temperatures (30 day) in each habitat.

**Table S1.** Species and geographical localities used in analyses (see Materials and Methods).
