# Supplementary figures and images for: Thermal tolerance and climate warming sensitivity in tropical snails
Source: Ecol Evol. 2015 Dec 2;5(24):5905–19. doi: 10.1002/ece3.1785 (PMC4717333; doi:10.1002/ece3.1785)

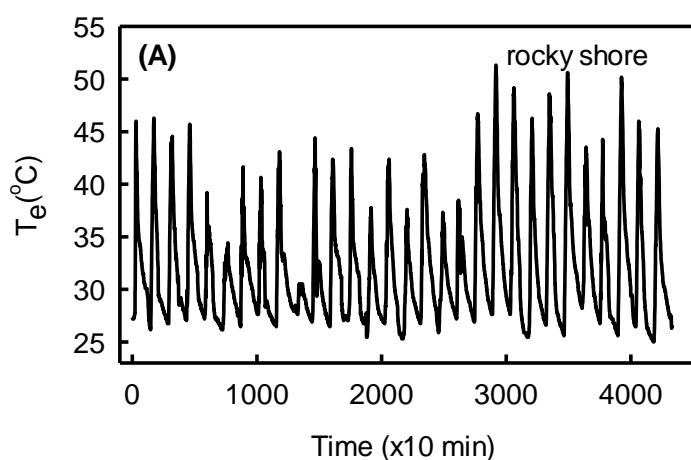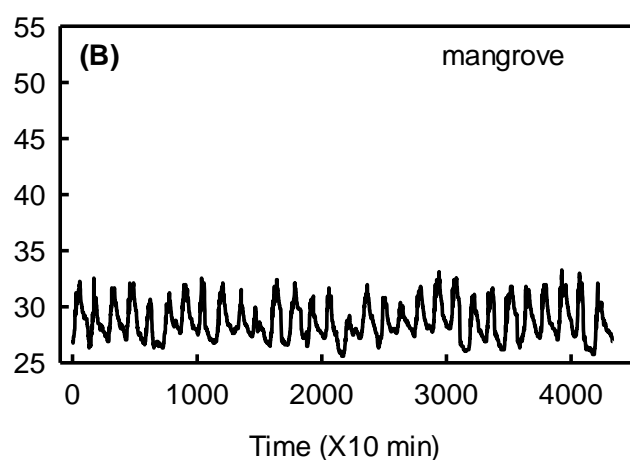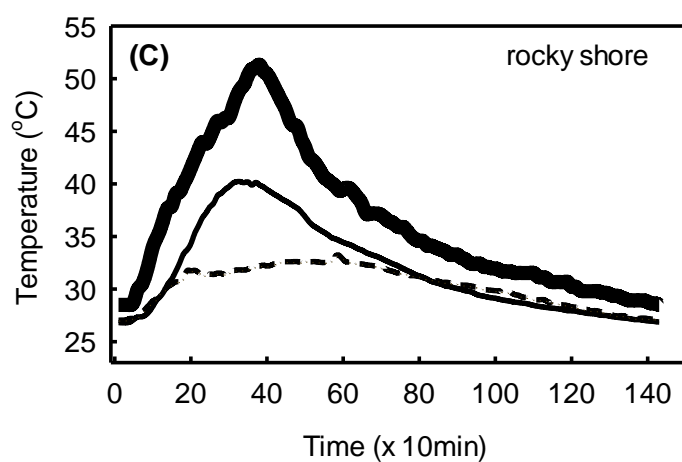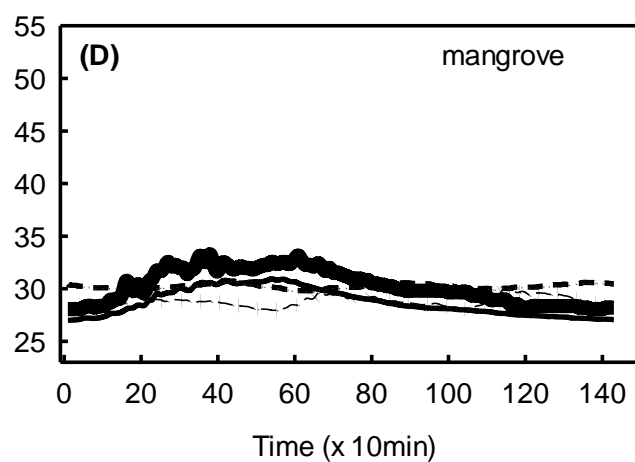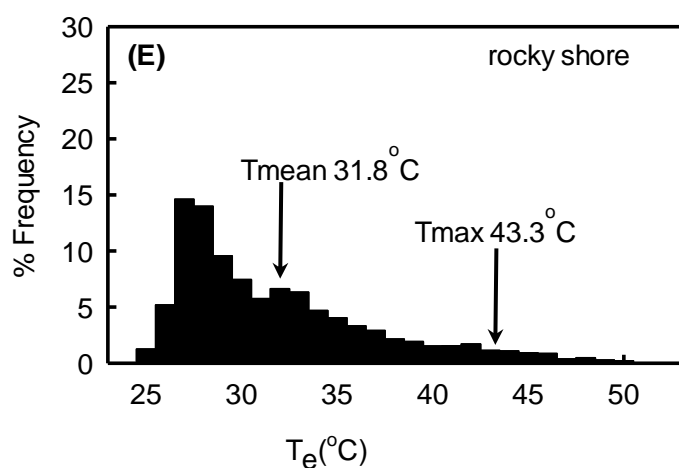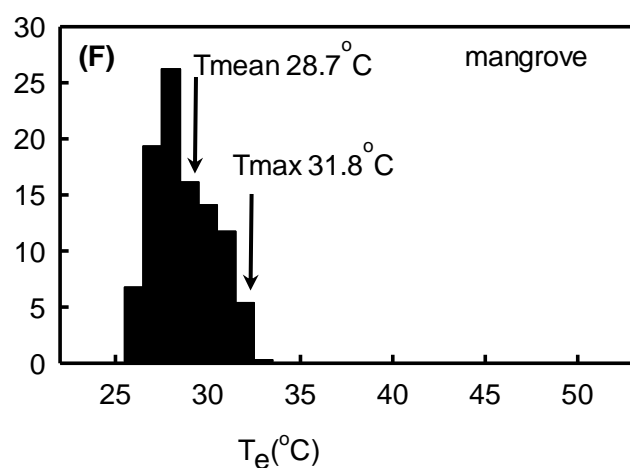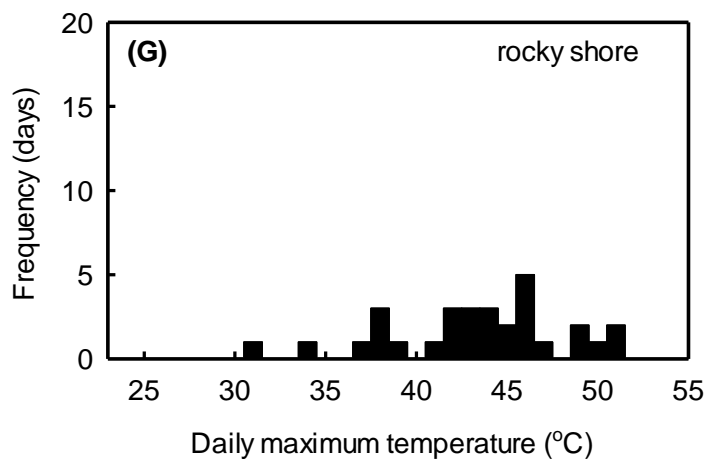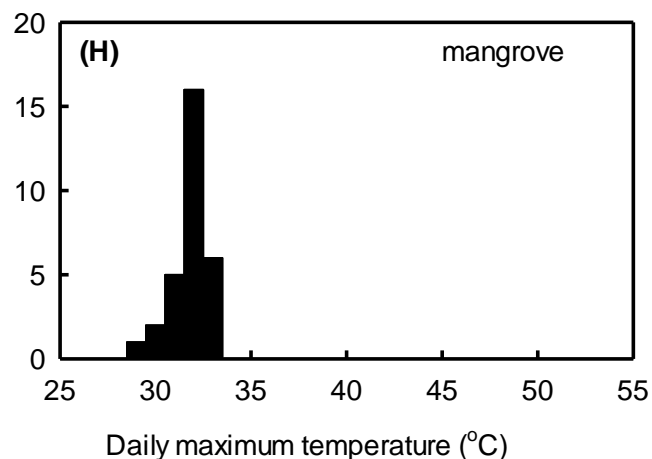

Supplement: Supplementary file 1 — Figure S1. Operative temperature data for sun‐exposed rocky shore (A, C, E, G) and shaded mangrove habitats (B, D, F, H). Mean temperatures for three habitats recorded every 10 min for 30 days (A, B), and frequency distributions of these means (E, F). (C, D) Average temperature for a 24 h period (0 and 144 = 06h00; 30 day) based on recordings in (A, B) are shown as thin solid lines, and for the hottest temperature in each habitat as thick solid lines. Dashed lines in the rocky shore panel indicate shaded (coolest) habitat, while dashed and dotted lines in the mangrove panel indicate temperatures on the mud surface (under tidal influence) or at the base of a tree, respectively. (G, H) Frequency distributions for daily maximum temperatures (30 day) in each habitat. [file ECE3-5-5905-s001.pdf]
